# In-Cell Proteomics Enables High-Resolution Spatial and Temporal Mapping of Early *Xenopus tropicalis* Embryos

**DOI:** 10.1016/j.mcpro.2025.101481

**Published:** 2025-12-05

**Authors:** Jian Sun, Xiaolu Xu, Shuo Wei, Yanbao Yu

**Affiliations:** 1 Department of Biological Sciences, University of Delaware, Newark, Delaware, USA; 2 Department of Chemistry and Biochemistry, University of Delaware, Newark, Delaware, USA

**Keywords:** data-independent acquisition, E4tip, in-cell proteomics, quantitative proteomics, *Xenopus tropicalis*, embryo, blastomere

## Abstract

Early embryonic development requires tightly regulated molecular programs to coordinate cell division, fate specification, and spatial patterning. While transcriptomic profiling has been widely performed, proteomic analyses of early vertebrate embryos remain limited owing to technical challenges in embryonic sample preparation. Here, we present an “in-cell proteomics” strategy, which bypasses cell lysis and yolk depletion, processes individual embryos directly in functionalized filter devices, and generates mass spectrometry (MS)-friendly samples in an extremely robust and streamlined manner. This single-vessel approach minimizes sample loss and technical variation, offering a highly sensitive and accurate alternative to low-input and low-cell quantitative proteomics. Coupled with field asymmetric ion mobility spectrometry and single-shot data-independent acquisition MS workflow, this approach enabled us to consistently quantify ∼6200 proteins from a single *Xenopus tropicalis* embryo, representing the deepest proteomic coverage of early *X. tropicalis* developmental stages reported to date. Investigation of the temporal proteomes across five cleavage stages (from 1- to 16-cell stages) revealed a drastic proteomic shift between 2- and 4-cell stages, followed by more gradual transitions thereafter. Spatial analysis of dissected 8-cell blastomeres uncovered pronounced molecular asymmetry along the animal–vegetal axis, whereas dorsal–ventral differences were minimal. This study establishes a novel in-cell proteomics technology in conjunction with field asymmetric ion mobility spectrometry and data-independent acquisition MS as a robust platform for high-resolution, low-input developmental proteomics analysis and provides a comprehensive spatiotemporal protein atlas for early *X. tropicalis* embryos.

## Highlights

• In-cell proteomics approach enables direct processing of proteins in the embryos.
• The approach offers high sensitivity and low variability for embryonic proteomics.
• Drastic proteome shift was revealed between 2- and 4-cell cleavage stages.
• Dissection of 8-cell blastomeres uncovered minimal dorsal–ventral differences.

## Graphical Abstract

**Figure:**
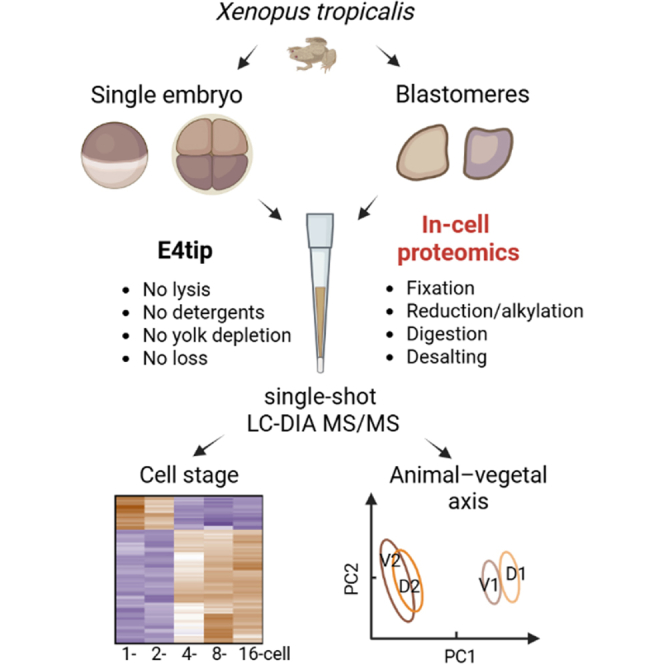

## In Brief

Conventional shotgun proteomic analysis of *Xenopus* embryos has been challenging due to tedious sample preparations, significant sample loss, and large variations. In this study, we developed an in-cell proteomics approach that can directly process proteins in the embryonic cells without lysis and extraction, offering the simplest approach reported to date for embryonic proteomics analysis. Applied to single *Xenopustropicalis* embryos and blastomeres, the approach showcased great potential for studying proteome-wide dynamics of early cleavage stages and dissecting dorsal–ventral differences.

Early vertebrate embryogenesis is driven by a series of precisely orchestrated molecular and cellular events that progressively specify cell fates and establish the body plan (1, 2). In amphibians, such as *Xenopus*, as in other vertebrates, this process begins with rapid, synchronous cleavage divisions that generate blastomeres with distinct developmental potentials along the animal–vegetal and dorsal–ventral axes (3, 4, 5). *Xenopus tropicalis*, a relative of the more widely used allotetraploid *Xenopus laevis*, is smaller—about one-third the body size and one-fifth the embryonic material (6). Due to its shorter generation time and true diploid genome, *X. tropicalis* has emerged as an excellent model for early vertebrate development (7, 8). While transcriptional regulation during early embryogenesis has been extensively studied (9), in particular using *X. laevis* embryos, the proteomic landscape of *X. tropicalis* remains largely unexplored, mainly because of challenges by the minute amounts of starting material and technical difficulties in sample preparation. For instance, the presence of abundant yolk proteins in every embryonic cell has severely hindered the detection of low-abundance proteins in *Xenopus* embryos (10, 11, 12). Although yolk depletion methods have been developed to improve mass spectrometry (MS) detection, these procedures are time consuming and labor intensive (10). Therefore, there are urgent unmet needs to develop effective and robust ways to examine the proteomes of *X. tropicalis* embryos at various developmental stages with low or no technical barriers.

Recent innovations in low-input proteomics, particularly the on-filter in-cell (OFIC) processing–based E4technology, have begun to overcome these limitations (13, 14). This single-vessel format technology enables direct *in situ* protein digestion within methanol-fixed cells, the so-called “in-cell proteomics,” thus greatly reducing sample handling and loss, and simplifying the proteomics workflow. This method has been shown to achieve unbiased and in-depth proteome coverage in yeast, mammalian cells, and *Caenorhabditis elegans* (13, 14). Importantly, the E4technology is compatible with low-cell or low-input applications, offering a practical solution for developmental studies where starting material is inherently limited. In the present study, we leveraged OFIC processing to analyze the temporal and spatial proteome dynamics of early *X. tropicalis* embryos. Specifically, we investigated spatiotemporal proteomes spanning five cleavage stages (1-cell to 16-cell stages) and spatially distinct blastomeres at the 8-cell stage.

## Experimental Procedures

### Experimental Design and Statistical Rationale

The *Xenopus* embryos were collected with four biological replicates (n = 4) for each of the five cleavage stages. The dorsal and ventral blastomeres were obtained with five biological replicates (n = 5) for each blastomere. All the replicate samples were processed in parallel. The proteome quantitation was performed using Spectronaut software (version 19.2; Biognosis AG). The protein inference algorithm was set to IDPicker. The false discovery rates (FDRs) of peptides, proteins, and peptide spectrum matches were all set to 0.01. For statistical analysis, the cutoffs for multisample test and two-sample test were set to permutation FDR 0.05 and *p* value 0.05, fold change 1.5, respectively, unless otherwise specified.

### *X. tropicalis* Embryo Culture and Collection

Wild-type *X. tropicalis* frogs were purchased from National *Xenopus* Resource in Woods Hole, MA. Methods involving live animals were carried out in accordance with the guidelines and regulations approved and enforced by the Institutional Animal Care and Use Committees at the University of Delaware (AUP #1307-2025-0). For temporal proteomic analysis, embryos at five developmental stages—1-cell, 2-cell, 4-cell, 8-cell, and 16-cell—were collected. Four embryos (biological replicates) per stage were collected. For spatial proteomic analysis, 20 embryos at the 8-cell stage were harvested and fixed in 100% methanol. Embryos were then manually dissected into four lineage-representative blastomeres: D1 (dorsal-animal), V1 (ventral-animal), D2 (dorsal-vegetal), and V2 (ventral-vegetal). For each blastomere group, five individually dissected cells were pooled to form one biological replicate, and five replicate digestion experiments were performed for each group.

### In-Cell Proteomics Sample Preparation

For in-cell sample preparation, individual embryos or blastomeres were collected and transferred to E4tips XL (CDS Analytical), which were prefilled with 200 μl of LCMS-grade methanol, and incubated on ice for 30 min. The tips containing fixed embryos were spun at 1500*g* for 1 min. The filters were washed once with 200 μl of methanol. Then 100 μl of 50 mM triethylammonium bicarbonate (TEAB) with final concentration of 10 mM Tris (2-carboxyethyl) phosphine and 40 mM chloroacetamide was added, followed by incubation at 45 °C for 10 to 15 min. The E4tips were spun and then washed once with 200 μl 50 mM TEAB. For protein digestion, 150 μl 50 mM TEAB plus 1.0 μg of trypsin/Lys-C mix (Promega) was added to the samples and incubated at 37 °C for 16 to 18 h with gentle shaking (350 rpm). After digestion, the samples were acidified with 1% formic acid (final concentration) and spun at low speed (400–600*g*) for 10 min. The tips were washed once with 200 μl of wash buffer (0.5% acetic acid in water) to discard flow through. The tips were transferred to new collection tubes and subjected to two sequential elution with 200 μl of elution buffer I (60% acetonitrile [ACN] and 0.5% acetic acid in water) and elution buffer II (80% ACN and 0.5% acetic acid in water), respectively. The elution was pooled, dried in the SpeedVac, and then stored at −80 °C until further analysis.

For SDS lysate–based digestion, a previously published protocol was followed with minor changes (15). Briefly, the embryos were first lysed with SDS buffer (4% SDS, 100 mM Tris–HCl, pH 8), vortexed at 1200 rpm for 5 min, sonicated with water bath for 3 min, and then boiled at 95 °C for 5 min. The lysate samples were mixed with 4x volume of 80% ACN and then transferred to E3tips (CDS Analytical) followed by centrifugation at 3000 rpm for 2 min. The tips were washed twice with 200 μl of 80% ACN and then incubated with final concentration of 10 mM Tris (2-carboxyethyl) phosphine and 40 mM chloroacetamide at 45 °C for 10 to 15 min. The following washing and digestion steps were similar to the E4tip procedure described above. After digestion, the E3tips were acidified with 1% formic acid (final concentration) and then stacked onto C18 based Stage tips (CDS Analytical) that were preactivated with methanol and equilibrated with 0.5% acetic acid in water. The stacked tips were then centrifuged at 1500 rpm for 10 min, followed by a wash step using 200 μl of 0.5% acetic acid in water and a quick centrifugation at 3000 rpm for 2 min. The resulting peptides were eluted sequentially with 200 μl of elution buffer I and II as described above. The eluted peptides were dried in SpeedVac, and stored the same way as described above.

### LC–MS/MS and Proteome Quantitation

The LC–MS/MS analysis was performed using an Ultimate 3000 RSLCnano system in conjunction with an Orbitrap Eclipse mass spectrometer and field asymmetric ion mobility spectrometry (FAIMS) Pro Interface (Thermo Scientific). The peptides resuspended in LC buffer A (0.1% formic acid in ACN) were first loaded onto a trap column (PepMap100 C18,300 μm × 2 mm, 5 μm particle; Thermo Scientific) and then separated on an analytical column (PepMap100 C18, 50 cm × 75 μm i.d., 3 μm; Thermo Scientific) at a flow of 250 nl/min. A linear LC gradient was applied from 1% to 25% mobile phase B (0.1% formic acid in ACN) over 125 min, followed by an increase to 32% mobile phase B over 10 min. The column was washed with 80% mobile phase B for 5 min, followed by equilibration with mobile phase A for 15 min. For MS analysis, the detector type was Orbitrap with resolution of 60,000; precursor MS range (*m/z*) was 380 to 980; automatic gain control target was standard; maximum injection time mode was auto. For MS/MS analysis, the instrument was operated under data-independent acquisition (DIA) mode. The isolation mode was quadruple; DIA window type was auto, and isolation window (*m/z*) was 8 with an overlap of 1; activation type was high-energy collisional dissociation with fixed collision energy mode (30%); the detector type was Orbitrap with a resolution of 15,000; normalized automatic gain control target (%) was 800, and the maximum injection time mode was auto; and the loop control was 2 s. For FAIMS compensation voltage setting, a 3-compensation voltage combination (−40, −55, and −75) was applied.

The MS raw data were processed using Spectronaut software (version 19.4) and a library-free DIA analysis workflow with directDIA+ and the *X. tropicalis* protein database (UniProt 2024 release; 76,225 sequences). Detailed parameters for Pulsar and library generation include trypsin/P as specific enzyme with minimum peptide length of seven amino acids and maximally two missed cleavages; toggle N-terminal M turned on; oxidation on M and acetyl at protein N terminus as variable modifications; carbamidomethyl on C as fixed modification; FDRs at peptide spectrum match, peptide and protein levels all set to 0.01; quantity MS level set to MS2, and cross-run normalization turned on. For mass tolerance, the calibration search was set to dynamic, and correction factors were set to one for both MS1 and MS2, which will allow Spectronaut to automatically calculate the optimal mass tolerances based on the data with no corrections applied. Bioinformatics analyses including *t* test, correlation, volcano plot, and clustering analyses were performed using Perseus software (version 2.1.0) (16) and GraphPad Prism (version 10), unless otherwise indicated.

## Results

### Temporal Profiling of Early *X. tropicalis* Embryo Proteomic Dynamics

**Fig. 1:**
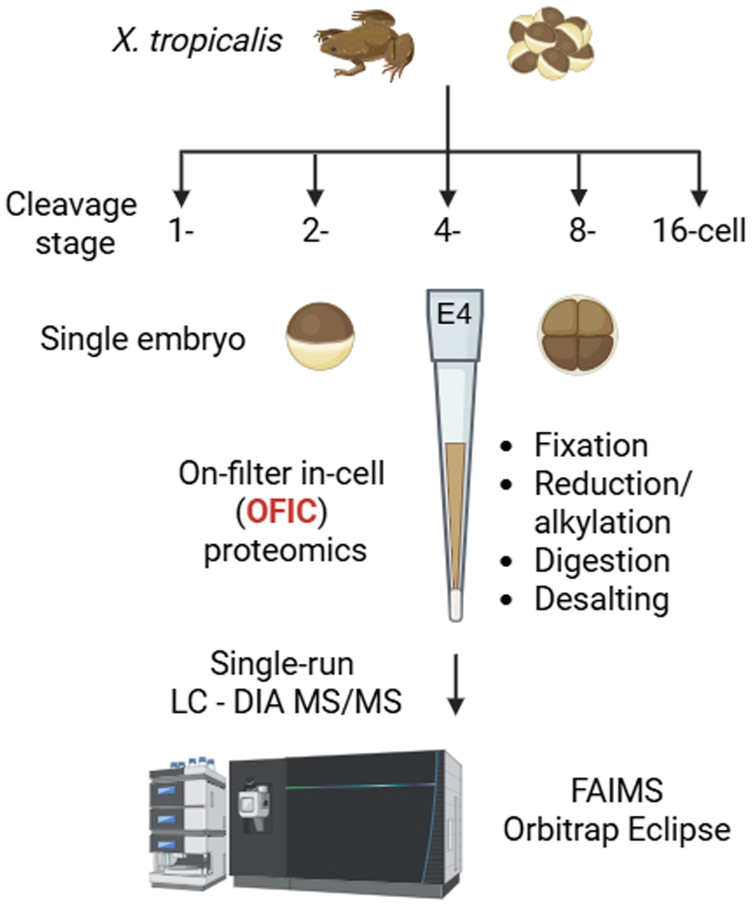
**Workflow of the “in-cell proteomics” approach for *Xenopus tropicalis* embryo analysis.** Embryos were collected at 1-, 2-, 4-, 8-, and 16-cell stage, respectively. One embryo was used for each digestion experiment, and four embryos (biological replicates) were processed independently for each cleavage stage using E4tip. Mass spectrometric analysis was performed using Orbitrap Eclipse with FAIMS Pro Interface in data-independent acquisition mode. FAIMS, field asymmetric ion mobility spectrometry.

Our first experiment was to investigate if the in-cell proteomics is applicable to embryonic samples. We collected individual *X. tropicalis* embryos and loaded directly to the filter device, E4tips, and then fixed the whole embryo with methanol. The fixed embryos were then subjected to tryptic digestion after reduction and alkylation in the cells (Fig. 1). With no fractionation or yolk depletion, we were able to identify over 5300 proteins from a single *X. tropicalis* embryo. Compared with conventional SDS-based lysis method, the in-cell digestion not only yielded consistently higher number of proteins and peptides identified but also greatly reduced variabilities (Supplemental Fig. S1, *A*–*D*). Regarding protein subcellular localization, we did not observe significant differences between the two processing strategies in the context of broad cellular compartments (such as nucleus, cytosol, mitochondrion, Golgi, and endoplasmic reticulum [ER]) as well as any particular membrane protein categories (Supplemental Fig. S1*E*). Encouraged by these initial results, we set out to further investigate how we can leverage this method to answer biologically significant questions.

**Fig. 2:**
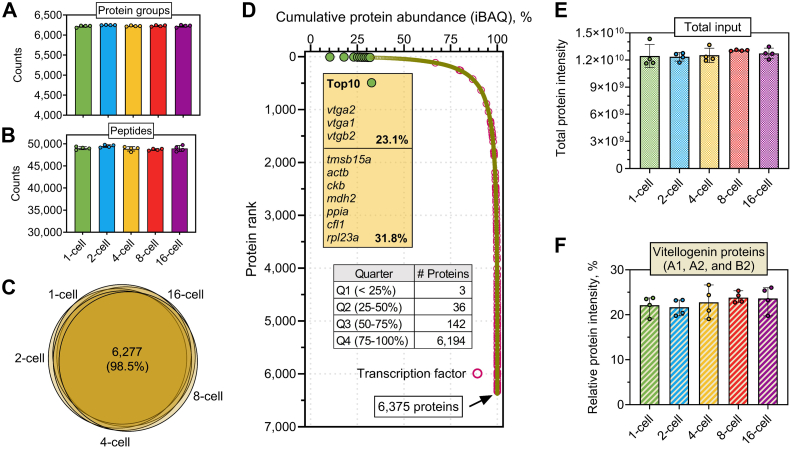
**Evaluation of the in-cell proteomics approach for *Xenopus tropicalis* embryo proteomic analysis.***A* and *B*, identification of protein groups and peptides across the five cleavage stages. Error bars indicate four biological replicates. *C*, Venn diagrams. The number and percentage indicate the shared proteins among the five stages. *D*, protein rank plot. The top 10 most abundant proteins were highlighted in *green* in the curve and were indicated in the text box. Transcription factors are highlighted in *orange circles*. The inner table shows the number of proteins in each quarter. *E*, total protein input of the five stages. The combined intensities of all the identified proteins derived from each stage were plotted. *F*, percentage of yolk proteins among the five stages. The intensity of three major vitellogenin proteins (A1, A2, and B2) were summed and divided by the overall protein intensity of each stage. Error bars indicate four biological replicates in this study.

We next analyzed the temporal proteome of the early cleavage stages, from 1- to 16-cell, with four biological replicates per stage following the workflow as described in Figure 1. Importantly, here we designed a larger version of E4tip (named E4tip XL), which can maximally alleviate the potential clogging issue by the large size of *Xenopus* embryos (approximately 0.5 mm in diameter). This in-cell proteomics approach enabled consistent identification from each one of the 20 digests (Fig. 2, *A* and *B*), resulting in a total of 6375 nonredundant protein groups. Among them, 6277 proteins were detected at all five stages (Fig. 2*C*), suggesting large qualitative similarities among the early embryonic proteomes. To our best knowledge, this study represents the largest *X. tropicalis* embryonic proteome reported to date (Supplemental Table S1). During the cleavage stages in the early *Xenopus* embryos, the successive cell divisions result in progressively smaller blastomeres, as the overall embryo volume remains largely constant (17, 18). This is reflected in the MS by the high consistency of the overall protein intensities (Fig. 2*E*, and Supplemental Fig. S2*A*). As a result, it ensured equivalent sample loading and proteome-wide label-free quantitation among the five cleavage stages. Based on the Xenbase annotation (19), the proteins also included 331 transcription factors (Fig. 2*D* and Supplemental Table S1), which are known to play crucial roles in early development but tend to have relatively low abundance. The frog embryo proteome spanned over seven orders of magnitude (Supplemental Fig. S2*C*). The three yolk precursor proteins—vitellogenin-A2, -A1, and -B2, ranked top three among the entire proteome and accounted for approximately 23% of the total protein mass, and also remained largely constant across the five developmental stages (Fig. 2*F*). The 39 most abundant proteins alone contributed to nearly half of the embryonic proteome (Fig. 2*D*). These data underscore the high dynamic range of embryonic proteome and the extreme challenge for low-abundance protein analysis. Previously, it was believed that the yolk proteins can make up >90% of the total proteome in the embryos, and yolk depletion has been exercised to enhance proteome coverage (10). Our findings suggested that the in-cell proteomics approach and the single-run DIA LC–MS can provide unprecedented proteomic depth, eliminating the need for yolk depletion or peptide prefractionation.

**Fig. 3:**
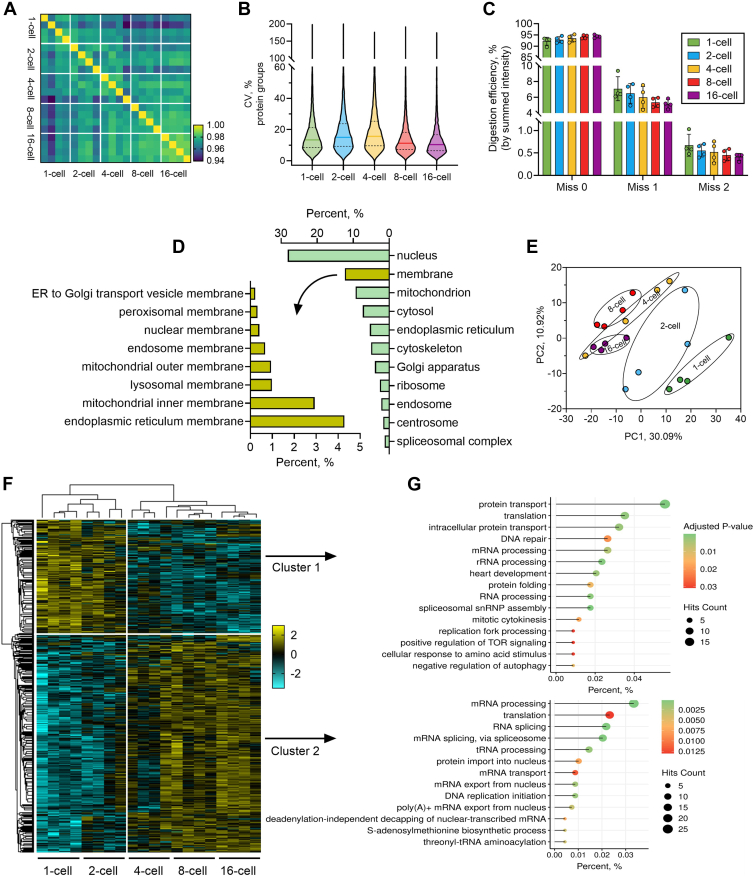
**Proteomics analysis of early embryos.***A*, Pearson’s correlation analysis. *B*, coefficient of variation of the protein groups among the five stages. *Horizontal solid lines* indicate the median value. *C*, digestion efficiency. The summed intensity of peptides carrying one and two missed cleavages were divided by the total peptide intensity. *D*, cellular compartment analysis of the overall *Xenopus tropicalis* embryo proteome. *E*, principal component analysis. *F*, heatmap of the ANOVA significant (false discovery rate = 0.05) proteins among the five stages. Two clusters were highlighted. Z-scored protein intensity values were plotted. *G*, Gene Ontology biological process analysis of proteins in cluster 1 and cluster 2, respectively.

In terms of technical performance of the OFIC approach, among the biological replicates at each stage, the Pearson's correlation averaged from 0.98 to 0.99, and the median of the protein group coefficient of variation ranged from 10% to 16% (Fig. 3, *A* and *B*), indicating excellent reproducibility of the approach. On the other hand, as the enzymatic penetration and in-cell digestion might be restricted by the embryo texture, we assessed the trypsin digestion efficiency by examining the number of peptides carrying uncleaved sites as well as their intensity. Our data implicate that although around 22% of the total number of peptides contained one or two miscleavages (Supplemental Fig. S2*D*), they accounted for 8% or less of the total peptide intensity (Fig. 3*C*), similar to lysate-based digestion experiments reported before (13). Although it might be reasonable to assume that more complex structures during embryonic development may become more resistant to proteolytic digestion, we did not observe significant variations of digestion efficiency among the five stages we studied here. This finding agrees well with our recent studies, where we used the OFIC approach to digest several different types of hard-to-lyse samples, such as *C. elegans* worms (14), plant leaf and pollen (20), sheath-forming bacteria (21), and yeast cells (22). We achieved excellent in-cell digestion efficiency, although these samples have either tough cuticle, strong cell walls, or durable outer layer that are highly resistant to cell lysis and protein digestion. Overall, these data suggest that in-cell digestion of *Xenopus* embryos after a simple methanol fixation is feasible.

Subcellular localization analysis (Fig. 3*D*) revealed a broad representation of cellular compartments, with the majority of proteins mapped to the nucleus (28%), membrane (12%), mitochondrion (9.4%), and cytosol (7.3%). Additional enrichment was observed in the ER, ribosome, Golgi apparatus, and endosome, indicating comprehensive proteome coverage across diverse cellular compartments. When compared with *X. laevis* data derived from a lysate-based digestion method (23), the proteins derived from OFIC approach did not show significant bias toward any particular cellular localization (Supplemental Fig. S3*A*). This finding is consistent with the data from in-cell digestion of *C. elegans* worms reported recently (14). Notably, the membrane proteins were not under-represented in the categories of mitochondrial inner and outer membrane proteins, ER membrane proteins, nuclear membrane proteins, and a few other organelles, consistent with our initial assessment data (Supplemental Fig. S1*E*). Interestingly, our data indicated that the OFIC approach is able to cleave and identify the transmembrane sequences of the membrane proteins, in addition to their cytosolic regions (Supplemental Fig. S3*B*). This finding is also supported by the identification of transmembrane proteins from plant leaves, which are extremely resistant to in-cell digestion (20). One explanation is that methanol solubilizes phospholipid bilayer, thus making the transmembrane regions exposed and accessible to proteolytic enzymes. Overall, these data demonstrated the unbiased performance of the in-cell digestion approach for global proteome analysis.

Next, we examined the proteome-wide differences among the five developmental stages. A simple principal component analysis using the global proteome profiles without imputation can already classify the developmental stages (Fig. 3*E*). While there were partial overlaps among 4-, 8-, and 16-cell-stage embryos, a clear segregation can be seen between the 2- and 4-cell stages, indicating a major proteomic transition during the early developmental window and a more gradual trajectory of proteomic changes at later cleavage stages. Multisample test (ANOVA, permutation FDR = 0.05) corroborated the findings and revealed over 1000 differential proteins and two distinct clusters between 1- and 2-cell (cluster 1) and 4/8/16-cell (cluster 2) stages (Fig. 3*F*), highlighting a key transition in the temporal progression of the early embryo proteome. The 363 proteins upregulated at earlier stages (cluster 1) were enriched in pathways related to protein metabolism, such as protein translation, folding, and transport, likely reflecting the earliest embryos’ reliance on maternal proteins and proteins translated from maternal mRNAs prior to the maternal to zygotic transition (Fig. 3*G*). Conversely, the 703 proteins upregulated at later stages (cluster 2) were enriched in pathways related to RNA metabolism, including RNA processing, transport, and translation, suggesting the activation of RNA regulatory pathways to support prezygotic transcription (Fig. 3*G*). While there was some functional overlap between the two clusters, such as enrichment in translation and DNA replication, these categories likely reflect distinct subsets of proteins undergoing turnover *versus* production. Taken together, the temporal proteomic profiles highlight a tightly regulated, stage-specific remodeling of the embryonic proteome, with the 2- to 4-cell transition representing a major developmental inflection point marked by dynamic proteomic reprogramming and fine-tuned regulation of core cellular processes.

### Spatial Quantitative Proteomic Characterization of 8-Cell Stage Blastomeres

**Fig. 4:**
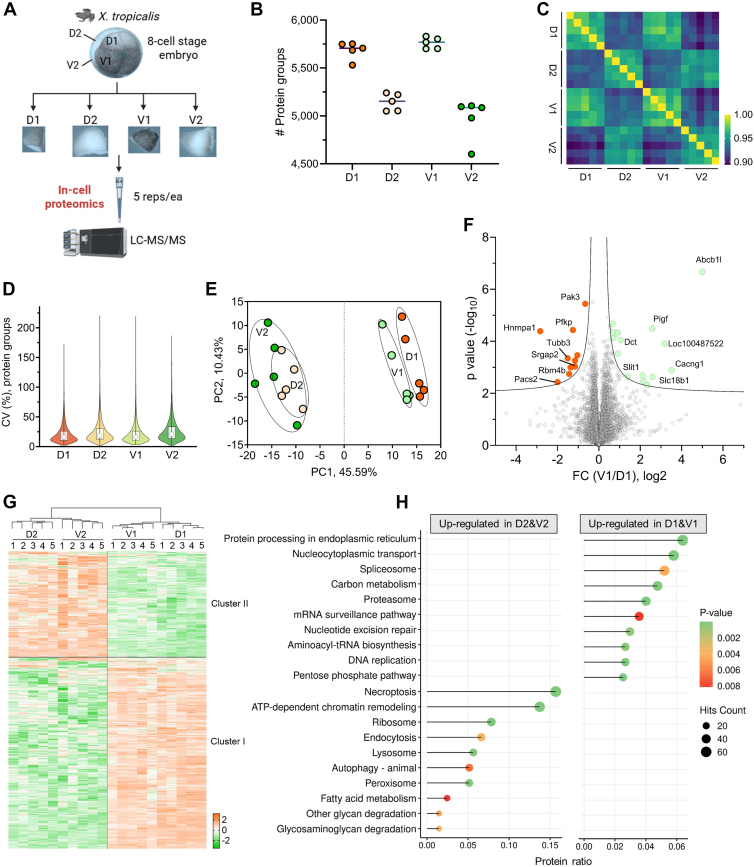
**Spatial proteomic profiling of 8-cell stage *Xenopus* tropicalis embryos using the OFIC approach.***A*, workflow of the OFIC approach for spatial proteomics analysis of 8-cell stage *X. tropicalis* embryos. *B*, protein group identifications. *C*, Pearson’s correlation. *D*, protein group coefficient of variation. *E*, principal component analysis. *F*, volcano plot of comparison between dorsal animal (D1) and ventral animal (V1) blastomeres. *Dotted lines* indicate permutation FDR of 0.05 and 0.01, respectively. *G*, heatmap of ANOVA significant proteins (FDR = 0.01). *H*, KEGG pathway analysis of the two cluster of proteins shown in *G*. Top 10 enriched terms of each cluster were plotted. FDR, false discovery rate; KEGG, Kyoto Encyclopedia of Genes and Genomes; OFIC, on-filter in-cell.

We further leveraged the E4technology to examine the individually dissected blastomeres from 8-cell stage embryos (Fig. 4*A* and Supplemental Table S2). The four types of blastomeres—D1 (dorsal-animal; future central nervous system [CNS]), V1 (ventral-animal; future neural crest and epidermis), D2 (dorsal-vegetal; future trunk mesoderm), and V2 (ventral-vegetal; future trunk endoderm) (24)—were obtained with five biological replicates per lineage (2 blastomeres per embryo; five embryos). Similar to whole embryo samples, the blastomeres were processed entirely in the E4tips (Fig. 4*A*). The streamlined workflow once again offered consistent identification rate (Fig. 4*B*), 5000 to 6000 protein hits from each replicate, and minimal variability, as demonstrated by low coefficient of variation and high quantitative correlation (Fig. 4, *C* and *D*). Unsupervised hierarchical clustering analysis revealed distinct proteomic patterns between animal pole (D1 and V1) and vegetal pole (D2 and V2) blastomeres (Supplemental Fig. S4), highlighting spatially organized proteomic signatures. The principal component analysis further supported this separation, with principal component 1 (45.6%) capturing the animal–vegetal distinction and principal component 2 (10.4%) distinguishing between D1 *versus* V1 and D2 *versus* V2 (Fig. 4*E*). These findings indicate that spatially distinct proteomic programs are already established at the 8-cell stage. ANOVA test (Benjamini–Hochberg FDR = 0.01) reported 2024 significantly differential proteins and two major molecular asymmetries across the animal–vegetal axis (Fig. 4*G* and Supplemental Table S2). Cluster 1 encompassed 1277 proteins enriched in animal pole blastomeres (D1 and V1), which were significantly enriched in pathways associated with anabolic processes, such as DNA, RNA, and protein synthesis, processing, and quality control (Fig. 4*H*). In contrast, cluster 2 comprised 747 proteins upregulated in vegetal blastomeres (D2 and V2), which showed upregulation of pathways related to catabolic processes—including necroptosis, autophagy, endocytosis, lysosome, and peroxisome (Fig. 4*H*). These signatures are consistent with the known divergence between the two sides, with the animal pole displaying more proliferative activity and the vegetal pole providing the energy and building blocks for growth (25). A global comparison between dorsal (D1 + D2) and ventral (V1 + V2) blastomeres revealed minimal differences (Supplemental Fig. S5). Since D2 and V2 did not segregate distinctly (Fig. 4*E*), likely reflecting slow development on the vegetal side, we focused on the animal side and looked into the comparison of D1 with V1 blastomere (Fig. 4*F*). Our data showed a total of 26 differential proteins (FDR = 0.05; S0 = 0.1) between V1 and D1. Interestingly, among the 10 proteins enriched in D1, six proteins (Pak3, Rbm4b, Srgap2, Tubb3, Pacs2, and Hnrnpa1) are known to be required for CNS development in mammals (see *Discussion* section), suggesting that these proteins may have very early roles in regulating CNS fate determination. We also identified Dct, an enzyme required for melanin maturation (26), as one of the proteins enriched in V1, providing a possible explanation for the higher pigmentation of the ventral blastomeres than their dorsal counterparts on the animal side (Fig. 4*A*) (26). Taken together, these spatially distinct proteomic signatures underscore the early emergence of lineage-specific molecular programs along the animal–vegetal axis of the 8-cell embryo, and suggest that dorsal–ventral proteomic asymmetry is limited at this stage, in contrast to the pronounced animal–vegetal divergence.

## Discussion

In this study, we established an “in-cell proteomics” platform as a robust and sensitive approach for developmental proteomics analysis of early *X. tropicalis* embryos. This platform enables in-depth, reproducible protein quantification directly from single embryos and individual blastomeres without the need for yolk depletion or peptide fractionation, overcoming long-standing technical barriers in low-input embryonic proteomics. The in-cell processing strategy preserves sample integrity and minimizes protein loss, allowing consistent detection of over 6000 proteins per sample, including low-abundance transcription factors. As global proteomics studies of *X. tropicalis* embryos are challenging and rarely seen, this dataset represents the deepest proteomic coverage reported to date for early *Xenopus* development. The excellent reproducibility and minimal variability highlight its suitability for deep quantitative proteomics analysis even in highly yolk-rich embryonic systems. It is noteworthy that in this study, we analyzed the OFIC digests using FAIMS in conjunction with DIA LCMS, which may have contributed to the deep coverage of the *Xenopus* embryo proteome as well. For instance, FAIMS can reduce background noises (in particular, the singly charged ions), provide an orthogonal online gas-phase separation to classical LCMS, and has been shown to improve identification rate by 30% to 50% (27, 28). DIA MS, compared with data-dependent acquisition, generally offers better reproducibility, higher proteome coverage, and is known to benefit the identification of low-abundance proteins (29). A very recent study by Distler *et al.* (30) demonstrated that DIA can identify on average 80% more proteins and peptides over data-dependent acquisition in the context of human plasma proteomics. Because the OFIC method can generate LCMS-friendly samples in such a simple and rapid fashion with excellent yield and reproducibility, we speculate that it can lead to even deeper proteome coverage when the in-cell digests are coupled with top-of-the-line LC–MS/MS systems.

One of the most striking findings in our temporal proteomic analysis was the proteome burst between the 2- and 4-cell stages, when transcription is largely absent. A plausible mechanism is the embryo’s reliance on post-transcriptional control of maternal mRNAs, particularly the dynamic remodeling of poly(A) tail during the oocyte-to-embryo transition. In vertebrate and fly embryos, poly(A)-tail length is strongly coupled to translational efficiency during early cleavage stages, providing a rapid way to remodel protein output without new transcription. This coupling then diminishes around gastrulation as zygotic control takes over (31, 32). Genome-wide tail-profiling and ribosome-profiling studies further show that cytoplasmic polyadenylation and deadenylation of maternal transcripts can broadly reshape the translational landscape during egg activation and early embryogenesis, independent of changes in mRNA abundance (32, 33). Thus, coordinated tail-length changes can drive stage-specific bursts of translation for large cohorts of maternal mRNAs, consistent with the proteomic reprogramming we observed at the 2-to-4-cell transition.

Our spatial proteomic analysis of 8-cell stage blastomeres revealed clear molecular asymmetry along the animal–vegetal axis that aligns with known developmental roles. Animal blastomeres exhibited strong enrichment of biosynthetic pathways, including nucleocytoplasmic transport, protein processing in the ER, and ribosome biogenesis. These features are consistent with the higher cell division rate of animal cells and their commitment to ectodermal lineages, which require sustained transcriptional and translational activities (34). In contrast, vegetal blastomeres showed pronounced enrichment in catabolic and metabolic processes, including lysosome, peroxisome, endocytosis, and autophagy. These pathways likely facilitate the mobilization and conversion of yolk reserves into metabolic fuel and biosynthetic precursors. This is consistent with the known role of the vegetal pole as a nutrient reservoir and with its slower division rate during early development (34, 35). Collectively, these spatial patterns suggest that animal and vegetal blastomeres adopt distinct proteomic strategies to meet the lineage-specific demands of early development.

One particularly interesting finding was the enrichment of necroptosis-related proteins in vegetal blastomeres. While necroptosis is classically associated with regulated cell death, recent studies have implicated components of this pathway in broader cellular roles, including organelle remodeling, metabolic regulation, and stress responses (36, 37). In the context of yolk-rich vegetal cells, we speculate that necroptosis-related factors may be repurposed to mediate intracellular remodeling during yolk utilization or to help maintain homeostasis under nutrient-dense, oxidative conditions. These proteins may also play nonlethal roles in developmental signaling or proteostasis during early mesendodermal patterning. Although the precise functional significance remains unclear, their consistent upregulation in vegetal blastomeres highlights a potentially underappreciated facet of early embryonic metabolism that warrent further investigation.

Direct comparison of animal-pole blastomeres (D1 *versus* V1) at the 8-cell stage highlights a small, functionally coherent set of proteins enriched in D1 (future CNS), many of which have established roles in vertebrate neurodevelopment. For example, Pak3, a neuronal p21-activated kinase, supports dendritic spine maturation and cognition (38). Srgap2 regulates actin dynamics and neurite/spine morphogenesis and modulates the timing of cortical synaptic maturation (39, 40). Rbm4b, an RNA-binding protein, contributes to alternative splicing and translational control as neural lineages consolidate (41). Tubb3 encodes the class III β-tubulin that supports axon outgrowth and growth-cone behavior (42). Pacs2, a scaffold/trafficking factor at ER–mitochondria contact sites, has been linked to neuronal excitability and neurodevelopmental phenotypes (43). Hnrnpa1, a broadly expressed RNA-binding protein with prominent neuronal functions, coordinates mRNA processing and localization (44); its higher abundance in D1 is consistent with a model in which post-transcriptional control helps prepattern neural competence. In contrast, V1 (ventral-animal) shows enrichment of Dct (dopachrome tautomerase), a melanogenic enzyme required for eumelanin maturation (26). Enrichment of Dct, likely as a consequence of cortical rotation, offers a mechanistic explanation for the classic observation that early ventral blastomeres are more pigmented than dorsal counterparts, a hallmark used by researchers to define the D–V axis in *Xenopus* embryos (45).

While our proteomic analysis provides high-resolution, quantitative insight into early embryonic dynamics, assigning functional relevance to individual proteins remains challenging. Due to the lack of transcriptional activity at this early stage, the proteins we detected here are likely deposited maternally or translated from maternal mRNA. While transcriptomic studies have provided insights into maternal mRNA dynamics in detail, relatively few studies have characterized the roles of maternal proteins because of technical difficulties. Consequently, although we identified hundreds of differentially expressed proteins during the 2- to 4-cell transition and across 8-cell blastomeres, the biological relevance of these proteins remains largely unexplored because of limited prior studies at this developmental stage. Addressing this knowledge gap will require integrated approaches combining proteomics, transcriptomics, and functional assays to systematically link maternal protein dynamics to specific developmental outcomes.

Despite these limitations, our study provides a valuable resource and technological advance for the field of developmental biology. By enabling high-resolution, low-input proteomic profiling of whole embryos and dissected blastomeres without yolk depletion or peptide fractionation, our approach overcomes key barriers that have historically limited access to early stage proteomic data. The resulting spatiotemporal protein atlas provides novel insight into molecular transitions and lineage-specific specialization in *X. tropicalis* embryos. These data serve as a foundational reference for future functional studies and offer a benchmark for implementing developmental proteomics in other model organisms. In this regard, our study represents not only a technical advance but also a conceptual step toward understanding how early proteome dynamics help shape embryonic patterning.

## Data Availability

The MS raw files associated with this study have been deposited to the MassIVE server (https://massive.ucsd.edu/) with the dataset identifier MSV000097784.

## Supplemental Data

This article contains supplemental data.

## Conflict of Interest

Y. Y. is a named inventor on a patent application (PCT/US2023/020,215) for the E4technology developed in this study, which has been licensed exclusively to CDS Analytical LLC (Oxford, PA) through the University of Delaware. Other authors declare no competing interests.
