# Supplementary material for: In-Cell Proteomics Enables High-Resolution Spatial and Temporal Mapping of Early Xenopus tropicalis Embryos
Source: Mol Cell Proteomics. 2025 Dec 5;25(2):101481. doi: 10.1016/j.mcpro.2025.101481 (PMC12927051; doi:10.1016/j.mcpro.2025.101481)
Supplement: Supporting Information [file mmc3.pdf]

Supporting Information

In-cell Proteomics Enables High-Resolution Spatial and Temporal Mapping of Early *Xenopus tropicalis* Embryos

Jian Sun[1,#], Xiaolu Xu[1,#], Shuo Wei[1,*], Yanbao Yu[2,*]

1. Department of Biological Sciences, University of Delaware, Newark, DE 19711, USA

2. Department of Chemistry and Biochemistry, University of Delaware, Newark, DE 19711, USA

# Equal contribution

* Correspondance: Shuo Wei, swei@udel.edu; Yanbao Yu, yybyu@udel.edu

Supporting information available

Supplementary Figure S1. Comparison of on-filter in-cell (OFIC) digestion of *X. tropicalis* embryos with SDS-based lysis method.

Supplementary Figure S2. Assessment of the temporal proteome of *X. tropicalis* embryos.

Supplementary Figure S3. Further assessment of the in-cell digestion approach.

Supplementary Figure S4. Unsupervised hieratical clustering analysis of the spatial proteomes of 8-Cell stage blastomeres.

Supplementary Figure S5. Volcano plot of comparison between dorsal and ventral blastomeres.

Supplementary Protocol. In-cell digestion of Xenopus embryos using E4tips.

Supplementary Figures

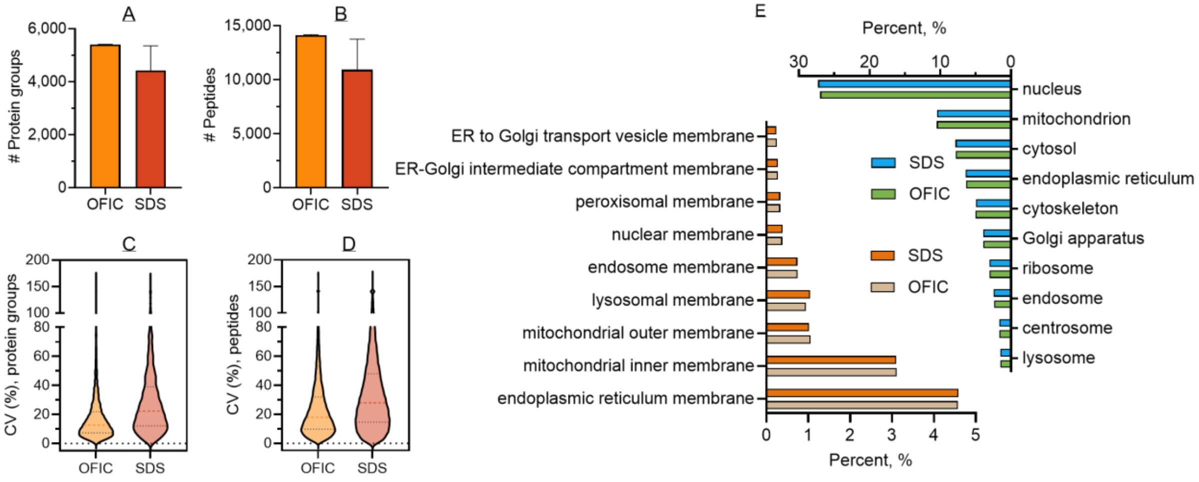

Supplementary Figure S1. Comparison of on-filter in-cell (OFIC) digestion of *X. tropicalis* embryos with SDS-based lysis method. (A-B) Protein and peptide identifications. Error bars represent three replicates. (C-D) Coefficient of variation of protein and peptide hits derived from the two methods. (E) Gene Ontology cellular compartment analysis of the proteins derived from the two methods. The membrane-related terms ($p < 0.01$) are shown on the left panel.

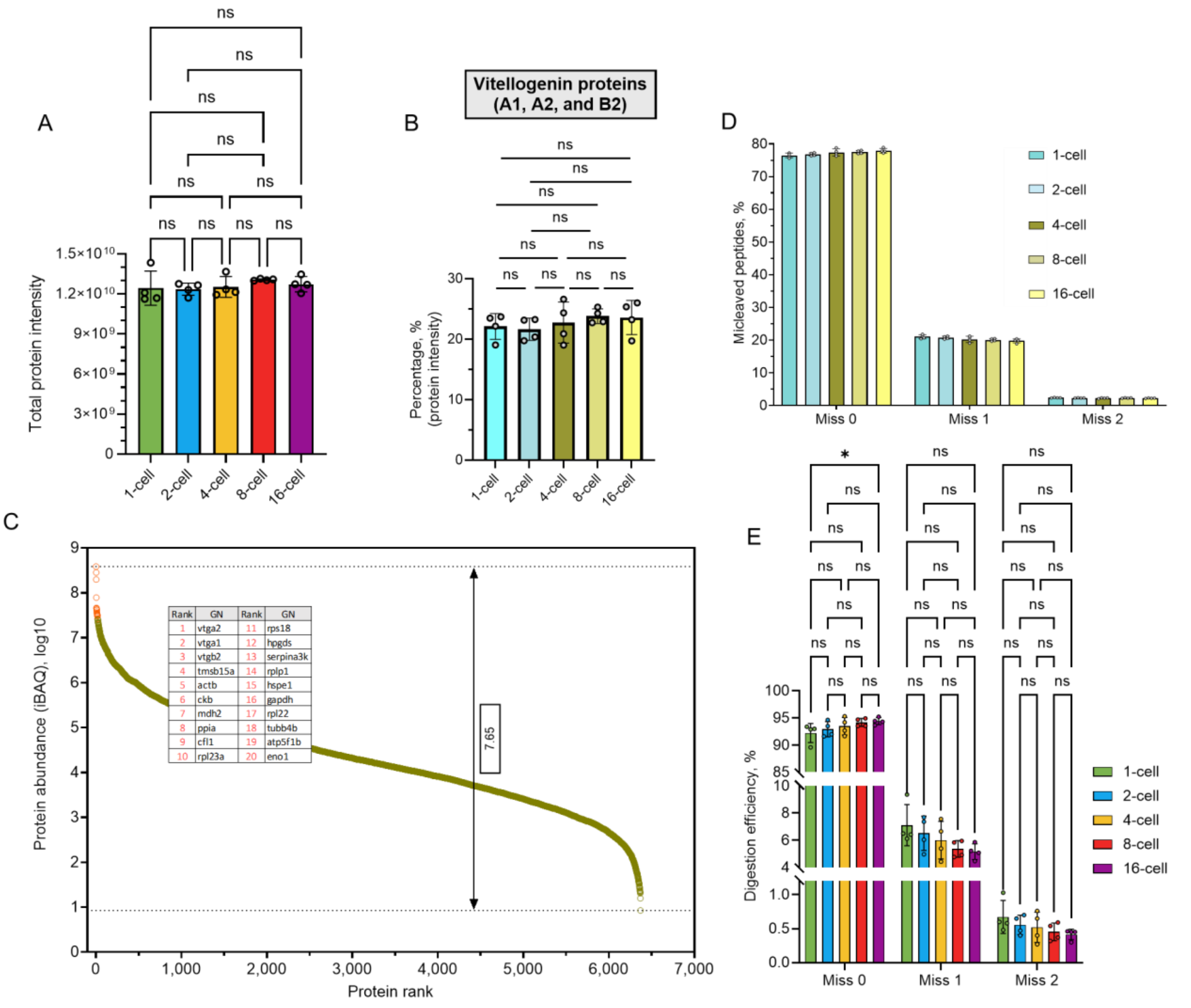

Supplementary Figure S2. Assessment of the temporal proteome of *X. tropicalis* embryos. (A) Plot of total protein intensity across the five stages. (B) Yolk protein contents among the five stages. (C) The complete protein rank plot. The top 20 most abundant proteins were indicated in the inner table. The straight arrow line indicates the dynamic range of embryo proteome. Median values (iBAQ, log10) of the embryonic proteins identified in the five stages were plotted. (D) Digestion efficiency assessed by the number of peptides that carry one or two missed cleavage sites. Significance (2-way ANOVA) of pair-wise comparisons was indicated.

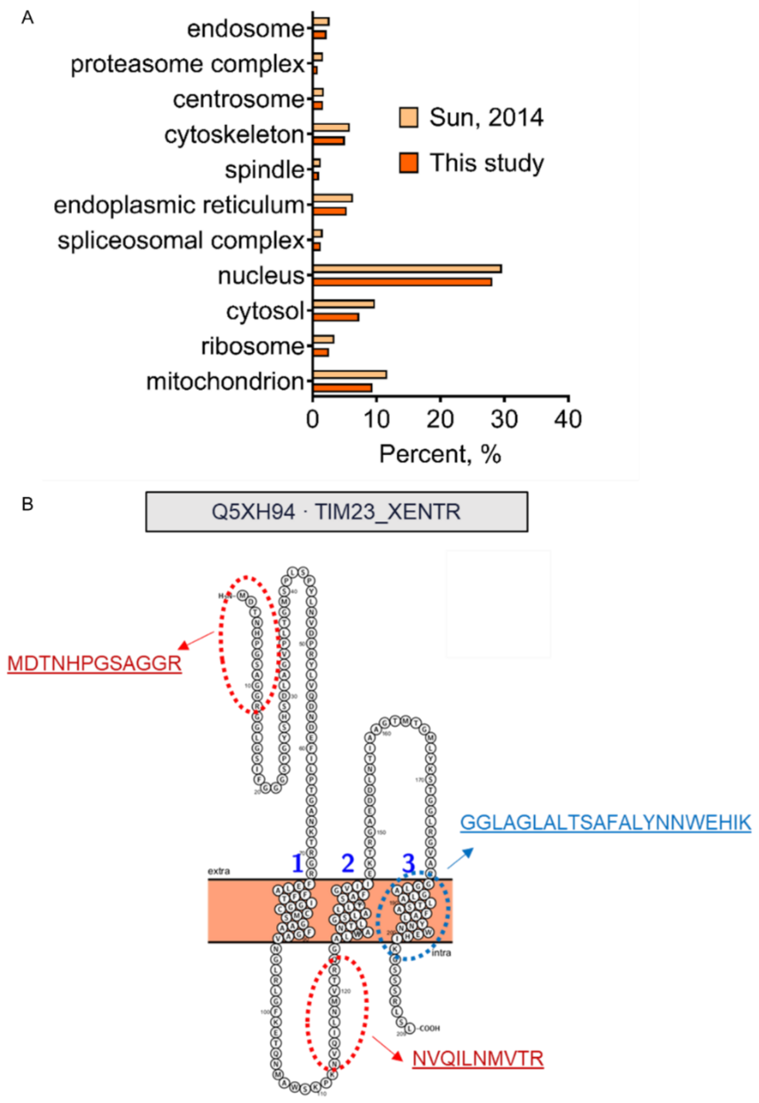

Supplementary Figure S3. Further assessment of the in-cell digestion approach. (A) Comparison of *Xenopus* proteins from two studies using Gene Ontology cellular compartment analysis (GOTERM_CC_DIRECT). For this study, the total 6,375 *X. tropicalis* proteins were used for the analysis. For the study by Sun *et al.* (Sci Rep. 2014, 4:4365. doi:10.1038/srep04365), the total 4,065 *X. laevis* proteins were used. (B) Illustration of a representative transmembrane protein identified by the OFIC approach. The identified peptide sequences were indicated in the image. Blue sequence indicates a tryptic peptide that locates in the transmembrane domain. The image was illustrated by Protter (http://wlab.ethz.ch/protter/start/). The transmembrane sequence information was obtained from UniProt knowledgebase (https://www.uniprot.org/uniprotkb/Q5XH94/entry).

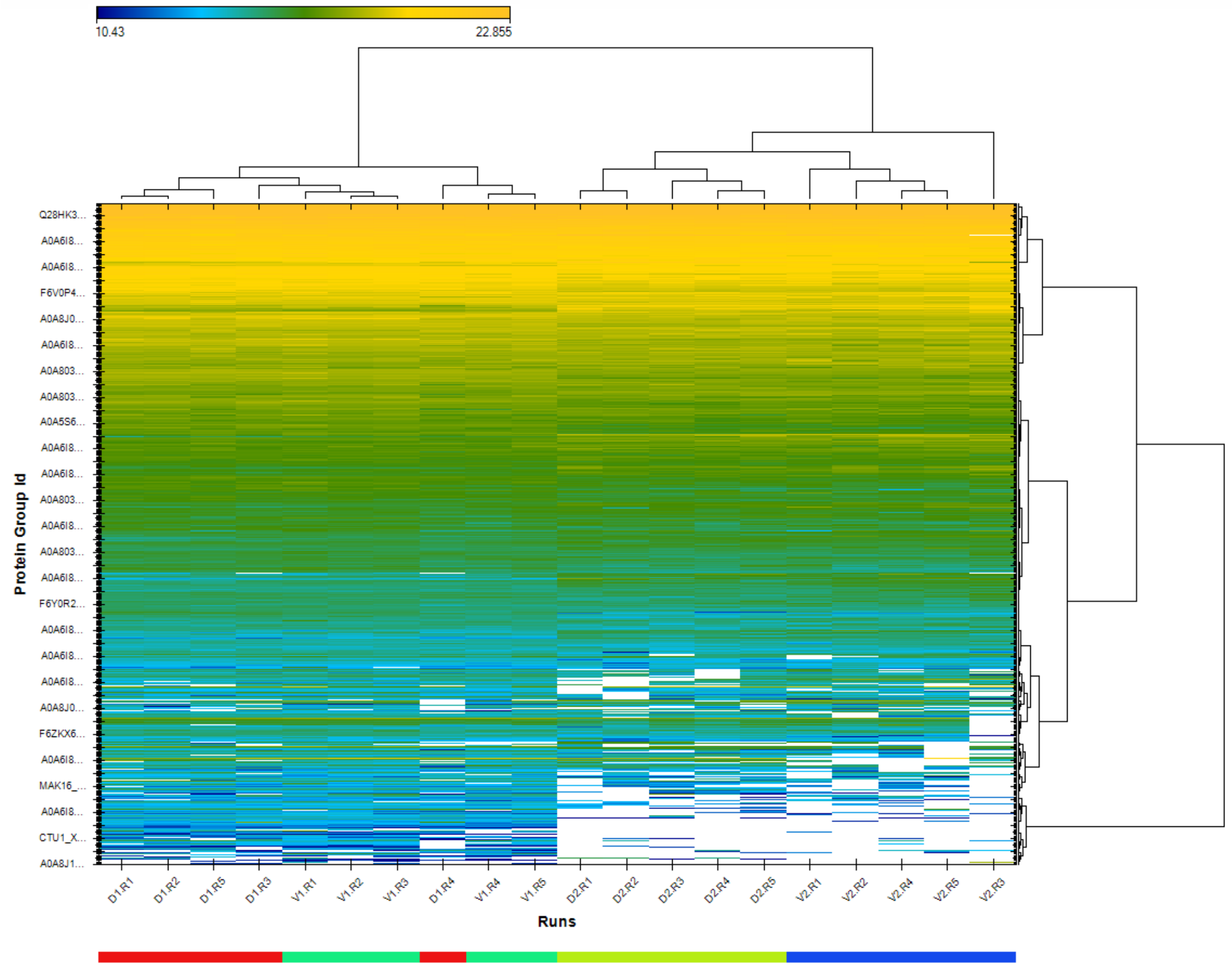

Supplementary Figure S4. Unsupervised hieratical clustering analysis of the spatial proteomes of 8-Cell stage blastomeres. Five biological replicates were processed for each blastomere. White boxes indicate missing values.

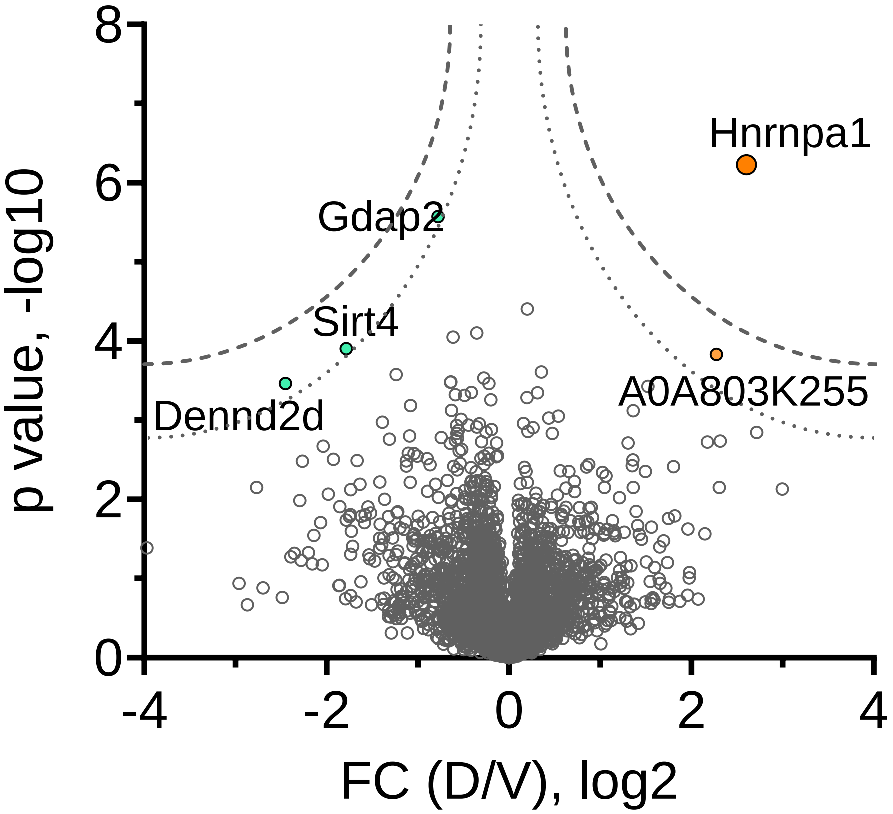

Supplementary Figure S5. Volcano plot of comparison between dorsal and ventral blastomeres. Dotted lines indicate Permutation FDR 0.05 and 0.01, respectively.

In-cell digestion of *Xenopus* embryos using E4tips

1. **Sample loading**
   Collect frog eggs, manually transfer one embryo to one E4tip via pipette.

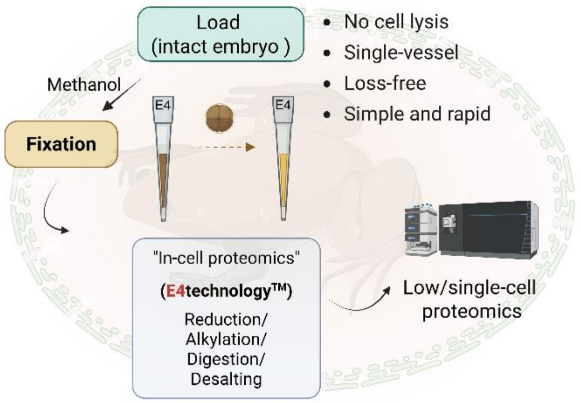

2. **Methanol fixation**
   Add 200 µl methanol, incubate at room temperature for 15 min. Centrifuge at 4,000 rpm for 2 min, discard flow through. Add 200 µl of methanol, repeat this step one more time.
   Tip: the flow through may be collected here for metabolomics analysis.
3. **Reduction and alkylation**
   Add final concentration of 10 mM Tris(2-carboxyethyl)phosphine (TCEP) and 40mM chloroacetamide (CAA) in 100 µl of 50 mM triethylammonium bicarbonate (TEAB), incubate at 45°C for 10-15 min with gentle shaking (300-500 rpm).
4. **Wash**
   Centrifuge at 4,000 rpm for 1 min to eliminate liquid. Add 200 µl of 50 mM TEAB solution, centrifuge again, and discard flow through.
5. **Digestion**
   Add 150 µl 50 mM TEAB, desired enzyme (Trypsin or Trypsin/Lys-C mix) at 1:50 ratio. Incubate at 37°C for 16-18 hours with gentle shaking. For one *Xenopus tropicalis* embryo, 1 µg of enzyme is suggested.
   Tip 1: please make sure no air gap between the buffer and the membrane filter. Do a pulse spin if desire (i.e., 2,000 rpm for 2 seconds).
   Tip 2: No caps are required for E4tips during overnight incubation.
6. **Acidification and desalting**
   After digestion, add formic acid to final concentration of 1%, centrifuge at 1,500 rpm for 10 min. Add 200 µl 0.5% acetic acid in water, centrifuge at 4,000 rpm for 2 min, discard flow through.
7. **Elution**
   Transfer E4tips to clean collection tubes, do two sequential elution by adding 200 µl 60% acetonitrile/0.5% acetic acid in water (elution I), and 80% acetonitrile/0.5% acetic acid in water (elution II), respectively; centrifuge at 4,000 rpm for 2 min to collect eluants to the same tube. Dry samples in the SpeedVac, and store at -80°C. The peptides are now desalted and ready for LCMS analysis.
